# Autism spectrum disorders

**DOI:** 10.4103/0256-4947.65261

**Published:** 2010

**Authors:** Hadeel Faras, Nahed Al Ateeqi, Lee Tidmarsh

**Affiliations:** a Developmental Pediatric Unit, Department of Pediatrics, Al-Sabah Hospital, Ministry of Health, Kuwait; b Department of Child Psychiatry, Montreal Children’s Hospital, McGill University Health Centre, Montreal, Quebec, Canada

## Abstract

Pervasive developmental disorders are a group of neurodevelopmental disorders characterized by impairments in communication, reciprocal social interaction and restricted repetitive behaviors or interests. The term autism spectrum disorders (ASD) has been used to describe their variable presentation. Although the cause of these disorders is not yet known, studies strongly suggest a genetic basis with a complex mode of inheritance. More research is needed to explore environmental factors that could be contributing to the cause of these disorders. The occurrence of ASD has been increasing worldwide, with the most recent prevalence studies indicating that they are present in 6 per 1000 children. The objectives of this article are to provide physicians with relevant information needed to identify and refer children presenting with symptoms suggestive of ASDs to specialized centers early, and to make them feel comfortable in dealing with public concerns regarding controversial issues about the etiology and management of these disorders.

Autism is one of a group of neurodevelopmental disorders known as pervasive developmental disorders (PDD). These disorders are characterized by three core deficits: impaired communication, impaired reciprocal social interaction and restricted, repetitive and stereotyped patterns of behaviors or interests. The presentation of these impairments is variable in range and severity and often changes with the acquisition of other developmental skills.

In 1943, the American psychiatrist Leo Kanner used the term “early infantile autism” to describe children who lacked interest in other people.^1^ In 1944, an Austrian pediatrician, Hans Asperger, independently described another group of children with similar behaviors, but with milder severity and higher intellectual abilities. Since then, his name has become attached to a higher functioning form of autism, Asperger syndrome.^2^ It was not until the 1980s that the term pervasive developmental disorders was first used.

The definition and diagnosis of these disorders has been broadened over the years to include milder forms of autism. The term autism spectrum disorders (ASDs) is currently used to describe three of the five pervasive developmental disorders listed in the Diagnostic and Statistical Manual of Mental Disorders, Fourth Edition (DSM-IV) and the International Classification of Diseases, Tenth Edition (ICD-10): autistic disorder, Asperger disorder and pervasive developmental disorder-not otherwise specified (PDD-NOS), Table 1.^3^,^4^

**Table 1:** The five pervasive developmental disorders.

Autistic Disorder Asperger disorder Rett disorder Childhood disintegrative disorder Pervasive developmental disorder- not otherwise specified (PDD-NOS)

A diagnosis of autistic disorder is made when there are impairments in communication and reciprocal social interaction with the presence of restricted repetitive and stereotyped patterns of behaviors or interests, prior to the age of 3 years. When autistic symptoms are present with no significant general delay in language and cognitive development, a diagnosis of Asperger disorder is made. A diagnosis of PDD-NOS is given when the triad of symptoms is present but the criteria are not met for a specific PDD.^3^ Often the label “high-functioning autism” is used interchangeably with Asperger disorder.^5^ This is controversial and there is considerable debate as to whether children with Asperger disorder, who have normal language milestones, should be considered to comprise a subgroup distinct from high-functioning children with autism, who have a history of delayed language development.^6^ The other two PDD’s, Rett syndrome and childhood disintegrative disorder, are rare and are associated with significant developmental regression, which makes them more distinct than the other disorders in the PDD group.^3^

## Epidemiology

ASD occurs more often in boys than girls, with a 4:1 male-to-female ratio.^7^ The reported prevalence rates of autism and its related disorders have been increasing worldwide over the past decades, from approximately 4 per 10 000 to 6 per 1000 children.^8^–^12^ The reasons for this increase include wider public awareness of these disorders, broadening of the diagnostic concepts, reclassifications of disorders and improved detection.^7^,^13^ The possibility that the increase in the reported cases is a result of unidentified risk factor(s) cannot be ruled out, and therefore more research is needed to address this.

## Etiology

The exact cause of autism and the other ASDs is still not known. The etiologic theories have changed over the years. It was once thought to be the result of faulty child-rearing. This historical psychosocial theory has been rejected, as research clearly indicates that the etiology is multi-factorial with a strong genetic basis.^14^ Although the etiology is not clear, there are a minority of cases, less than 10%, where autism is part of another condition. Such cases are often referred to as “secondary” autism; these include tuberous sclerosis, fragile X syndrome, phenylketonuria and congenital infections secondary to rubella and cytomegalovirus.^14^–^20^

## Genetic factors

Family studies have demonstrated that autism is both familial and heritable. The recurrence rate in siblings of an autistic child is 2% to 8%, which is higher than that of the general population.^8^,^17^ Furthermore, twin studies showed that monozygotic twins have a higher concordance rate than dizygotic twins—90% and 10%, respectively.^14^,^15^,^17^ Other genetic studies suggest a complex mode of inheritance, with linkage studies suggesting genetic loci on several chromosomes including chromosome 7^17^ and chromosome X.^15^

## Environmental factors

Various environmental factors have been explored as possible causative agents in autism. Epidemiological studies indicate that some environmental factors, including prenatal infections with rubella and cytomegalovirus, account for few cases of autism.^17^ The role of heavy metals in the etiology of autism is controversial and requires more research.^21^

There are public concerns about vaccines being possible triggers for autism. There have been two separate hypothesis relating vaccination to autism, the first is the potential adverse effect of the measle, mumps, rubella (MMR) vaccine and the second is that of thimerosal, a mercury-based preservative used in some vaccines. These two hypotheses are distinct from each other; MMR vaccines have never contained thimerosal, because it would inactivate a live vaccine.^7^

## Measles, mumps and rubella vaccine and the ASD hypothesis

An article published in 1998 suggested a possible relationship between the MMR vaccine and ASD. Since then, there has been a decline in the rate of MMR vaccination among children.^22^,^23^ As a consequence of that, there have been measles outbreaks.^23^ These factors led to the conduction of large worldwide studies to examine this potential relationship. These studies showed that there is no association between MMR vaccine and ASDs.^24^–^29^ The measles vaccine has been proven to be safe and effective in preventing this potentially lethal disease. Therefore, with the lack of scientific evidence that MMR vaccine is causally related to autism, the administration of MMR vaccine should be encouraged to prevent measles outbreak.

## Thimerosal and the ASD hypothesis

Thimerosal is a mercury-containing compound that has been used as an additive to vaccines to prevent bacterial contamination. In 1997, the United States Food and Drug Administration called for assessment of the risk of all mercury-containing foods and drugs. This action stimulated the United States Public Health Service and the American Academy of Pediatrics to issue a joint statement in 1999 calling for the removal of thimerosal from the vaccines. This action was undertaken as a precautionary measure. There was no evidence that mercury was harmful at the doses being administered in the vaccines.^30^ The evidence from these studies does not support and favors rejection of a causal relationship between thimerosal-containing vaccines and autism.^28^,^31^–^34^ Further evidence contradicting this hypothesis is that rates of autism have continued to increase despite the removal of thimerosal from vaccines in 1999.^7^,^33^

## Diagnosis

Physicians play an important role in early recognition of ASD, because they are usually the first point of contact for parents. Therefore, it is important that physicians be able to recognize the various signs and symptoms of this group of disorders.^35^ Physicians should be alerted to the possibility of autism and its related disorders when there are qualitative impairments in social, language and communication skills, as well as repetitive interests and behaviors. The severity of these impairments varies significantly among children with ASD. Even though the typical age of onset is before 3 years, the impairments can be subtle and may not be detected before school age. An example of this are children with Asperger disorder, who may be identified and diagnosed much later than children with typical autism, on average at 11 years of age.^5^ This is because parents of children with Asperger disorder may not recognize the subtle abnormalities in their child’s behavior, because they may not have an opportunity to compare them to peers. Conversely, physicians and teachers compare a child’s behavior to typically developing children and notice abnormalities and impairments more easily.^5^ This reflects the importance of gathering information about the child from multiple sources, especially when diagnosing the broader pervasive developmental disorders.^5^,^36^

The diagnosis is challenging in children at the both ends of the spectrum. Children with severe autistic symptoms may be hard to differentiate from those with isolated severe intellectual disability, while those with mild symptoms may be misdiagnosed as having a language disorder or a social anxiety. Table 2 lists some of the general developmental warnings of possible ASD in children and adolescents.

**Table 2:** Red flags indicating possible autism spectrum disorder.*

Pre-school children	
Communication impairment	Delayed or absent speech Deficient nonverbal communication e.g. lack pointing, difficulty following a point
Social impairment	Lack of response to others’ facial expression/feeling Lack of pretend play; little or no imagination Lack of showing typical interest in or play near peers purposefully Lack of initiation of activity Inability to share pleasure
Impairments of interests, activities and/or behaviors	Unusual or repetitive hand and finger mannerism Liking sameness/inability to cope with change Repetitive play with toys (eg, lining up toys; turning lights on and off)

**School-age children**	
Communication impairment	Abnormalities in language development including muteness Persistent echolalia Unusual vocabulary for child’s age/social group
Social impairment	Inappropriate attempts at joint play (eg, may manifest as aggressive or disruptive behavior) Lack of awareness to classroom ‘norms’ (criticizing teachers, unwilling to cooperate in classroom activities)
Impairments of interests, activities and/or behaviors	Lack of flexible cooperative imaginative play/creativity Inability to cope with change Presence of odd behaviors including unusual response to sensory stimuli

**Adolescents:**	
Language, non-verbal skills and social communication	Problems with communication, even if wide vocabulary and normal use of grammar. May be unduly quiet, may talk at others rather than hold a to-and-fro conversation, or may provide excessive information on topics of own interest. Unable to adapt style of communication to social situations (eg, may sound like ‘a little professor’ (overly formal) or be inappropriately familiar. May have speech peculiarities including ‘flat’, unmodulated speech, repetitiveness, use of stereotyped phrases. May take things literally and fail to understand sarcasm or metaphor. Unusual use and timing of non-verbal interaction (eg, eye contact, gesture and facial expression)
Social problems	Difficulty making and maintaining peer friendships, though may find it easier with adults or younger children. Can appear unaware or uninterested in peer group ‘norms’, may alienate by behaviors which transgress ‘unwritten rules’. May lack awareness of personal space, or be intolerant of intrusions on own space.
Rigidity in thinking and behavior	Preference for highly specific, narrow interests or hobbies, or may enjoy collecting, numbering or listing. Strong preferences for familiar routines, may have repetitive behaviors or intrusive rituals Problems using imagination e.g. in writing, future planning. May have unusual reactions to sensory stimuli (eg sounds, tastes, smell, touch, hot or cold.

* From the Scottish Intercollegiate Guidelines Network (SIGN) 98.^45^

Several structured instruments have been used for screening for ASDs in high-risk children, e.g. siblings of autistic children, children with developmental delay or genetic syndromes. The Checklist for Autism in Toddlers (CHAT), Modified-Checklist for Autism in Toddlers (M-CHAT), Childhood Autism Rating Scale (CARS) and Social Responsiveness Scale-Parent and Teacher (SRS), are some of the widely used instruments for screening these high-risk children.^37^–^40^ Although these are well-structured instruments, they should be used with caution as they are screening tests and should not be used to rule out or confirm the diagnosis.

The diagnosis of ASD is best achieved by a team of health care professionals who are well informed and experienced in these disorders. Therefore, a primary care health worker should refer any child suspected of having ASD to a specialist team for detailed assessment. The specialist team makes the diagnosis of ASD based on an autism-specific history and clinical observation. Specific tools commonly used in the assessment include the Autism Diagnostic Interview-Revised (ADI-R) and the Autism Diagnostic Observational Schedule (ADOS).^41^–^43^

Most children with ASDs have a normal physical examination. The physical examination should include observations of any dysmorphic features or skin pigmentations along with a detailed neurological examination that may be suggestive of secondary causes, such as fragile X syndrome or tuberous sclerosis. Currently, there are no laboratory or radiologic tests to diagnose ASD. The diagnostic yield from biomedical investigations is low.^44^ Most guidelines and practice parameters on ASD recommend conducting DNA analysis for fragile X syndrome, regular karyotyping, audiology testing and other investigations where clinically relevant.^45^,^46^ The yield of an etiologic investigation may be increased with the presence of coexisting developmental delay or intellectual disability.^8^

## Treatment

ASDs are lifelong chronic disabilities. At present, there is no cure for the core symptoms of autism. However, several groups of medications, including atypical neuroleptics, have been used to treat associated behavioral problems such as aggression and self-injurious behaviors.^47^,^48^

Research has shown that the most effective therapy is use of early intensive behavioral interventions that aim to improve the functioning of the affected child. These interventions focus on developing language, social responsiveness, imitation skills, and appropriate behaviors. Examples of these behavioral therapies include ABA (Applied Behavior Analysis) and TEACCH (Treatment and Education of Autistic and Related Communication Handicapped Children). The ABA approach involves teaching new behaviors by explicit reinforcement; problem behaviors are addressed by analyzing triggers in order to change factors in the environment that are contributing to that behavior.^49^ The TEACCH approach takes advantage of relative strengths in visual information processing using strategies such as visual schedules, clearly structured and organized classrooms, and highly structured learning activities that are broken down into manageable, visually organized steps.^49^ These behavioral techniques should begin early in the pre-school period and be followed by highly individualized educational intervention in the school.^50^–^54^

Since conventional medicine has failed to find a cure for ASD, families have been seeking complementary and alternative therapies in search of a cure. These include the use of mega-vitamins and other nutritional supplements, chelation therapy and hyperbaric oxygen therapy. Currently, there is no evidence to support the use of nutritional supplements as an intervention for ASD.^21^,^55^,^56^ There have been claims that diets free of casein and/or gluten are effective interventions for autism. The latest Cochrane review in 2008 reported lack of evidence to support the use of these diets for children with ASD, and stated that there is a lack of research on the potential harms and disbenefits of these diets.^56^ Therefore, families who desire to try these diets should be counseled on the need for calcium and vitamin D supplements with attention to the protein intake, since milk and dairy products represent major sources of calcium and protein intake for these young children.

Despite the lack of scientific evidence, desperate and vulnerable parents seek chelation therapy for their affected children in an attempt to remove heavy metals that are thought to cause autism.^57^ The use of chelation therapy in children with autism and its related disorders has not been validated. Furthermore, it is an invasive procedure and can have fatal consequences.^57^ Recently, hyperbaric oxygen therapy has increased in popularity as an alternative therapy for ASDs.^58^ This therapy involves inhaling up to 100% oxygen in a pressurized chamber to increase plasma oxygen to the tissues including the brain.^58^,^59^ Because of the limited research in this field, conclusions about the efficacy of hyperbaric oxygen therapy as a treatment for children with ASDs cannot be drawn at this time.^58^–^60^

## Conclusion

ASDs are a group of disorders characterized by impairments in three domains, namely communication, reciprocal social interaction and behaviors that are restricted and repetitive in nature. Physicians play a crucial role in the early identification of children with these disorders since they are the first point of contact and the starting point for referral to appropriate centers for further evaluation and management. These disorders are increasing in prevalence, so that physicians are more likely to encounter them during their practice. Therefore, physicians need to be aware of the variable presentation of these disorders in order to identify affected children early and refer them appropriately to specialized centers for evaluation, counseling and intervention. Furthermore, physicians need to feel comfortable in dealing with public concerns regarding controversial issues about the etiology and management of these disorders.

## References

[1] Kolvin I (1971). Studies in childhood psychoses: I. Diagnostic criteria and classification. Brit J Psychiatry.

[2] Klin A (2003). Asperger syndrome: an update. Rev Bras Psiquiatr.

[3] (2000). American Psychiatric Association. Diagnostic and Statistical Manual of Mental Disorders.

[4] World Health Organization. International Statistical Classification of Diseases and Related Health Problems, 10th Revision.

[5] Mattila ML, Kielinen M, Jussila K, Linna SL, Bloigu R, Ebeling H, Moilanen I (2007). An epidemiological and diagnostic study of Asperger syndrome according to four sets of diagnostic criteria. J Am Acad Child Adolesc Psychiatry.

[6] McAlonan GM, Suckling J, Wong N, Cheung V, Lienenkaemper N, Cheung C (2008). Distinct patterns of grey matter abnormality in high-functioning autism and Asperger’s syndrome. J Child Psychol Psychiatry.

[7] Fombonne E, Zakarian R, Bennett A, Meng L, McLean-Heywood D (2006). Pervasive developmental disorders in Montreal, Quebec, Canada: prevalence and links with immunizations. Pediatrics.

[8] Chakrabarti S, Fombonne E (2001). Pervasive developmental disorders in preschool children. JAMA.

[9] Chakrabarti S, Fombonne E (2005). Pervasive developmental disorders in preschool children: confirmation of high prevalence. Am J Psychiatry.

[10] (2006). Centers for Disease Control and Prevention. Mental health in the United States: parental report of diagnosed autism in children aged 4-17 years, United States, 2003-2004. MMWR Morb Mortal Wkly Rep.

[11] Bertrand J, Mars A, Boyle C, Bove F, Yeargin-Allsop M, DeCoufle P (2001). Prevalence of autism in a United States population: the Brick Township, New Jersey, investigation. Pediatr.

[12] Yeargin-Allsopp M, Rice C, Karapurkar T, Doernberg N, Boyle C, Murphy C (2003). Prevalence of autism in a US metropolitan area. JAMA.

[13] Shattuck PT (2006). The contribution of diagnostic substitution to the growing administrative prevalence of autism in US special education data. Pediatr.

[14] Bailey A, Le Couteur A, Gottesman, Bolton P, Simonoff E, Yuzda E (1995). Autism as a strongly genetic disorder: evidence from a British twin study. Psychol Med.

[15] Marco EJ, Skuse DH (2006). Autism-lessons from the X chromosome. Soc Cogn Affect Neurosci.

[16] Kothur K, Ray M, Malhi P (2008). Correlation of autism with temporal tubers in tuberous sclerosis complex. Neurol India.

[17] Muhle R, Trentacoste SV, Rapin I (2004). The genetics of autism. Pediatrics.

[18] Bolton PF, Park RJ, Higgins JN, Griffiths PD, Pickles A (2002). Neuro-epileptic determinants of autism spectrum disorders in tuberous sclerosis complex. Brain.

[19] Trottier G, Srivastava L, Walker CD (1999). Etiology of infantile autism: a review of recent advances in genetic and neurobiological research. J Psychiatry Neurosci.

[20] Steiner CE, Acosta AX, Guerreiro MM, Marques-de-Faria AP (2007). Genotype and natural history in unrelated individuals with phenylketonuria and autistic behavior. Arq Neuropsiquiatr.

[21] Levy SE, Hyman SL (2008). Complementary and alternative medicine treatments for children with autism spectrum disorders. Child Adolesc Psychiatr Clin N Am.

[22] Wakefield AJ, Murch SH, Anthony A, Linnell J, Casson DM, Malik M (1998). Ileal-lymphoid-nodular hyperplasia, non-specific colitis and pervasive developmental disorder in children. Lancet.

[23] Jansen VA, Stollenwerk N, Jensen HJ, Ramsay ME, Edmunds WJ, Rhodes CJ (2003). Measles outbreaks in a population with declining vaccine uptake. Science.

[24] Kaye JA, del Mar Melero-Montes M, Jick H (2001). Mumps, measles, and rubella vaccine and the incidence of autism recorded by general practitioners: a time trend analysis. BMJ.

[25] Madsen KM, Hviid A, Vestergaard M, Wohlfahrt J, Thorsen P, Olsen J (2002). A population-based study of measles, mumps and rubella vaccination and autism. N Engl J Med.

[26] Dales L, Hammer SJ, Smith NJ (2001). Time trends in autism and in MMR immunization coverage in California. JAMA.

[27] Taylor B, Miller E, Lingam R, Andrews N, Simmons A, Stowe J (2002). Measles, mumps, and rubella vaccination and bowel problems or developmental regression in children with autism: population study. BMJ.

[28] Institute of Medicine of National Academies [Internet]. Immunization Safety Review: Vaccines and Autism. Institute of Medicine of National Academies: Immunization safety review committee. Academies 2004.

[29] DeStefano F, Bhasin TK, Thompson WW, Yeargin-Allsopp M, Boyle C (2004). Age at first measles-mumps-rubella vaccination in children with autism and school-matched control subjects: a population-based study in metropolitan Atlanta. Pediatr.

[30] (2007). Infectious Diseases and Immunization Committee, Canadian Paediatric Society. Autistic spectrum disorder: No causal relationship with vaccines. Paediatr Child Health.

[31] Hviid A, Stellfeld M, Wohlfahrt J, Melbye M (2003). Association between thimerosal-containing vaccine and autism. JAMA.

[32] Andrews N, Miller E, Grant A, Stowe J, Osborne V, Taylor B (2004). Thimerosal exposure in infants and developmental disorders: a retrospective cohort study in the United Kingdom does not support a causal association. Pediatrics.

[33] Madsen KM, Lauritsen MB, Pedersen CB, Thorsen P, Plesner AM, Andersen PH (2003). Thimerosal and the occurrence of autism: Negative ecological evidence from Danish population-based data. Pediatrics.

[34] Parker SK, Schwartz B, Todd J, Pickering LK (2004). Thimerosal-containing vaccines and autistic spectrum disorder: a critical review of published original data. Pediatrics.

[35] Johnson CP, Myers SM (2007). American Academy of Pediatrics Council on Children With Disabilities. Identification and evaluation of children with autism spectrum disorders. Pediatrics.

[36] Risi S, Lord C, Gotham K, Corsello C, Chrysler C, Szatmari P (2006). Combining information from multiple sources in the diagnosis of autism spectrum disorders. J Am Acad Child Adolesc Psychiatry.

[37] Baron-Cohen S, Wheelwright S, Cox A, Baird G, Charman T, Swettenham J (2000). Early identification of autism by the CHecklist for Autism in Toddlers (CHAT). J R Soc Med.

[38] Wong V, Hui LH, Lee WC, Leung LS, Ho PK, Lau WL (2004). A modified screening tool for autism (Checklist for Autism in Toddlers [CHAT-23]) for Chinese children. Pediatrics.

[39] Pereira A, Riesgo RS, Wagner MB (2008). Childhood autism: translation and validation of the childhood autism rating scale for use in Brazil. J Pediatr (Rio J).

[40] Charman T, Baird G, Simonoff E, Loucas T, Chandler S, Meldrum D, Pickles A (2007). Efficacy of three screening instruments in the identification of autistic-spectrum disorders. Br J Psychiatry.

[41] Rutter M, Le Couteur A, Lord C (2003). Autism Diagnostic Interview-Revised.

[42] Lord C, Risi S, Lambrecht L, Leventhal BL, DiLavore PC, Pickles A (2000). The autism diagnostic observation schedule-generic: a standard measure of social and communication deficits associated with the spectrum of autism. J Autism Dev Disord.

[43] Akshoomoff N, Corsello C, Schmidt H (2006). The Role of the Autism Diagnostic Observation Schedule in the Assessment of Autism Spectrum Disorders in School and Community Settings. Calif School Psychol.

[44] Barton M, Volkmar F (1998). How commonly are known medical conditions associated with autism?. J Autism Dev Disord.

[45] Scottish Intercollegiate Guidelines Network (SIGN) 98. Assessment, diagnosis and clinical intervention for children and young people with autism spectrum disorders. A nation clinical guideline.

[46] Filipek PA, Accardo PJ, Baranek GT, Cook EH, Dawson G, Gordon B (1999). The screening and diagnosis of autistic spectrum disorders. J Autism Dev Disord.

[47] McCracken JT, McGough J, Shah B, Cronin P, Hong D, Aman MG (2002). Risperidone in children with autism and serious behavioral problems. N Engl J Med.

[48] Canitano R, Scandurra V (2008). Risperidone in the treatment of behavioral disorders associated with autism in children and adolescents. Neuropsychiatr Dis Treat.

[49] Barbaresi WJ, Katusic SK, Voigt RG (2006). Autism: a review of the state of the science for pediatric primary health care clinicians. Arch Pediatr Adolesc Med.

[50] Ospina MB, Krebs Seida J, Clark B, Karkhaneh M, Hartling L, Tjosvold L (2008). Behavioural and developmental interventions for autism spectrum disorder: a clinical systematic review. PLoS One.

[51] Ooi YP, Lam CM, Sung M, Tan WT, Goh TJ, Fung DS (2008). Effects of cognitive-behavioural therapy on anxiety for children with high-functioning autistic spectrum disorders. Singapore Med J.

[52] Couper JJ, Sampson AJ (2003). Children with autism deserve evidence-based intervention. Med J Aust.

[53] Williams White S, Keonig K, Scahill L (2007). Social skills development in children with autism spectrum disorders: a review of the intervention research. J Autism Dev Disord.

[54] Sallows GO, Graupner TD (2005). Intensive behavioral treatment for children with autism: four-year outcome and predictors. Am J Ment Retard.

[55] Nye C, Brice A (2005). Combined vitamin B6-magnesium treatment in autism spectrum disorder. Cochrane Database Syst Rev.

[56] Millward C, Ferriter M, Calver SJ, Connell-Jones GG (2008). Gluten- and casein-free diets for autistic spectrum disorder. Cochrane Database Syst Rev.

[57] Baxter AJ, Krenzelok EP (2008). Pediatric fatality secondary to EDTA chelation. Clin Toxicol.

[58] Rossignol DA, Rossignol LW, James SJ, Melnyk S, Mumper E (2007). The effects of hyperbaric oxygen therapy on oxidative stress, inflammation, and symptoms in children with autism: an open-label pilot study. BMC Pediatr.

[59] Chungpaibulpatana J, Sumpatanarax T, Thadakul N, Chantharatreerat C, Konkaew M, Aroonlimsawas M (2008). Hyperbaric oxygen therapy in Thai autistic children. J Med Assoc Thai.

[60] Rossignol DA, Rossignol LW, Smith S, Schneider C, Logerquist S, Usman A (2009). Hyperbaric treatment for children with autism: a multicenter, randomized, double-blind, controlled trial?. BMC Pediatr.

